# Magnetic resonance imaging for detecting root avulsions in traumatic adult brachial plexus injuries: protocol for a systematic review of diagnostic accuracy

**DOI:** 10.1186/s13643-018-0737-2

**Published:** 2018-05-19

**Authors:** Ryckie G. Wade, Yemisi Takwoingi, Justin C. R. Wormald, John P. Ridgway, Steven Tanner, James J. Rankine, Grainne Bourke

**Affiliations:** 1 Department of Plastic and Reconstructive Surgery, Leeds Teaching Hospitals Trust, Leeds, UK; 2 Faculty of Medicine and Health Sciences, University of Leeds, Leeds, UK; 3 Institute of Applied Health Research, University of Birmingham, Birmingham, UK; 4 Department of Plastic and Reconstructive Surgery, Stoke Mandeville Hospital, Buckinghamshire Healthcare NHS Trust, Aylesbury, UK; 5 Nuffield Department of Orthopaedics, Rheumatology and Musculoskeletal Sciences (NDORMS), University of Oxford, Oxford, UK; 6 Department of Radiology, Leeds Teaching Hospitals Trust, Leeds, UK; 7 Leeds Biomedical Research Centre, Chapel Allerton Hospital, Leeds, UK

**Keywords:** Review, Diagnostic test accuracy, Root avulsion, Pre-ganglionic, Brachial plexus, Magnetic resonance imaging, Sensitivity, Specificity

## Abstract

**Background:**

Adult brachial plexus injuries (BPI) are becoming more common. The reconstruction and prognosis of pre-ganglionic injuries (root avulsions) are different to other types of BPI injury. Preoperative magnetic resonance imaging (MRI) is being used to identify root avulsions, but the evidence from studies of its diagnostic accuracy are conflicting. Therefore, a systematic review is needed to address uncertainty about the accuracy of MRI and to guide future research.

**Methods:**

We will conduct a systematic search of electronic databases alongside reference tracking. We will include studies of adults with traumatic BPI which report the accuracy of preoperative MRI (index test) against surgical exploration of the roots of the brachial plexus (reference standard) for detecting either of the two target conditions (any root avulsion or any pseudomeningocoele as a surrogate marker of root avulsion). We will exclude case reports, articles considering bilateral injuries and studies where the number of true positives, false positives, false negatives and true negatives cannot be derived. The methodological quality of the included studies will be assessed using a tailored version of the QUADAS-2 tool. Where possible, a bivariate model will be used for meta-analysis to obtain summary sensitivities and specificities for both target conditions. We will investigate heterogeneity in the performance of MRI according to field strength and the risk of bias if data permits.

**Discussion:**

This review will summarise the current diagnostic accuracy of MRI for adult BPI, identify shortcomings and gaps in the literature and so help to guide future research.

**Systematic review registration:**

PROSPERO CRD42016049702.

**Electronic supplementary material:**

The online version of this article (10.1186/s13643-018-0737-2) contains supplementary material, which is available to authorized users.

## Background

Traumatic brachial plexus injuries (BPI) in adults are common following road traffic collisions. In England, there are 48,000 cases of major trauma per annum [1] and 1% have a brachial plexus injury [2]. Such injuries can result in permanent disability [3–7], pain [8, 9], psychological morbidity [10, 11] and reduced quality of life [3, 5, 12]. With optimal reconstructive surgery, patients can recover useful function [3, 4, 12, 13] which is associated with improved quality of life [5].

**Fig. 1:**
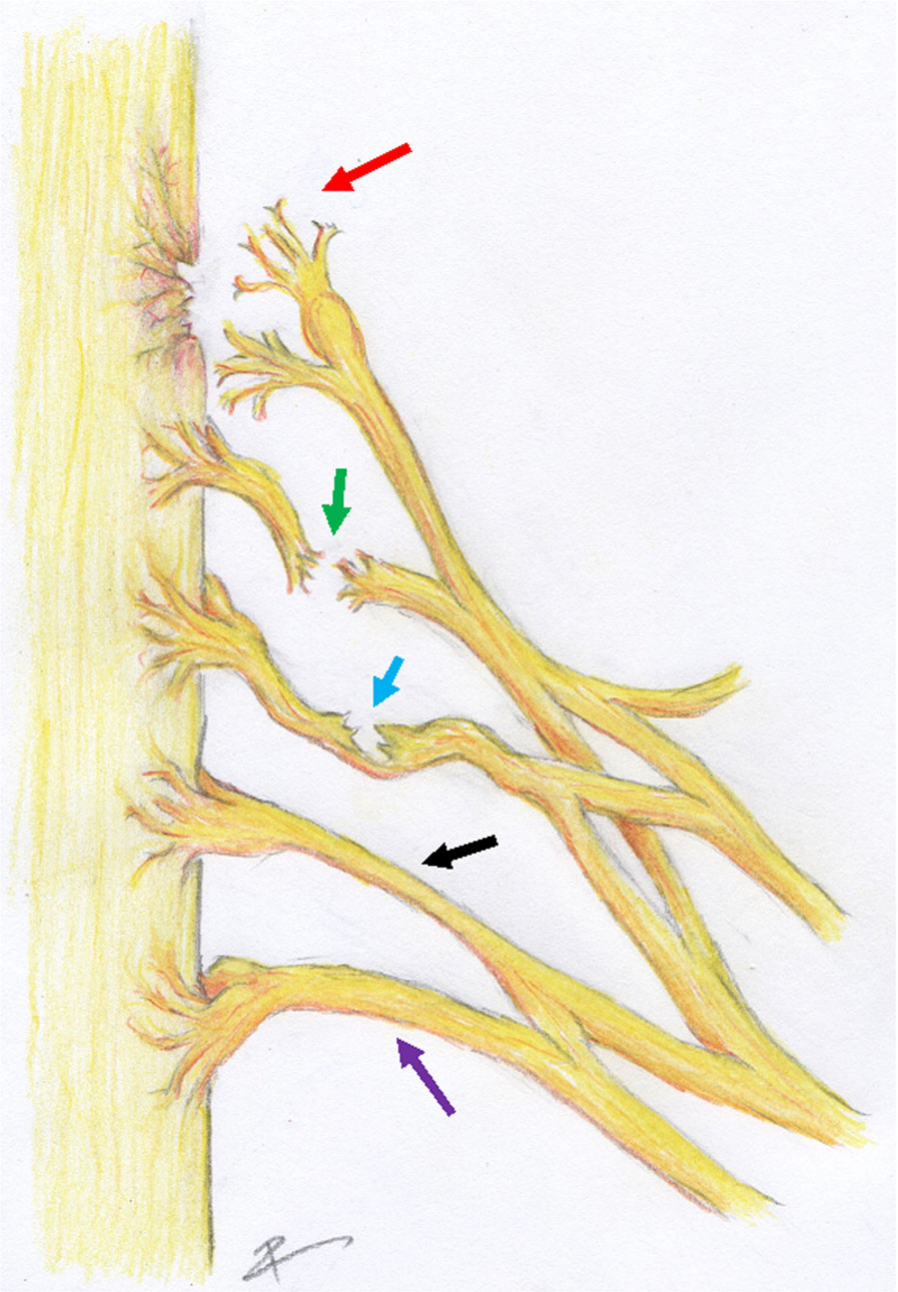
A schematic of the roots, trunks and divisions of the left brachial plexus. The red arrow illustrates a root avulsion of C5. The green arrow indicates a complete rupture of the C6 root. The blue arrow points to a partial rupture of the middle trunk. The black arrow indicates an attenuated C8 root in continuity. The purple arrow indicates a normal T1 root

To date, magnetic resonance imaging (MRI) is the best indicator of various pathologies affecting the brachial plexus [14], and in the context of trauma it is superior to pre-operative nerve conduction studies [15], high-resolution ultrasonography [16–18] and intraoperative somatosensory-evoked potentials [19]. However, MRI is still unable to differentiate nerve injuries which need reconstructive surgery and those which will recover spontaneously. Further, MRI is unable to differentiate post-ganglionic (Fig. 1; green, blue and black arrows) and pre-ganglionic (Fig. 1; red arrow) nerve injuries which is of paramount importance because their reconstruction and prognosis is different. Post-ganglionic nerve injuries (ruptures or attenuations) have a more favourable prognosis because the anterior horn cells in the spinal cord persist [20, 21]; therefore, if continuity can be re-established in a timely fashion, then motor recovery can be expected [22–30]. Conversely, reconstructing pre-ganglionic nerve injuries (known as root avulsions) requires nerve transfers as the cell bodies of the native motor neurones recede [5, 6, 13, 20] and re-implantation of roots remains of uncertain value [31–33]. Therefore, the identification of root avulsion(s) is critical as it alters the operative plan and prognosis.

Delay to surgical reconstruction is the leading cause of poor outcome [28, 29], and so, most surgeons use pre-operative MRI and neurophysiological tests [15] in an effort to identify those with avulsion injuries. However, the reliability of pre-operative MRI is uncertain, and therefore, most surgeons still undertake early surgical exploration to identify root avulsion(s). Numerous studies have examined the diagnostic accuracy of MRI for traumatic BPI, but few have specifically considered root avulsion injuries and fewer still had an adequate reference standard, as many used clinical follow-up (i.e. the reanimation of the limb) [34] or electrophysiological studies [35, 36] as surrogate markers. Therefore, it is unsurprising that the literature is conflicting on the ‘overall accuracy’ of MRI for traumatic root avulsion.

Our rationale for conducting this review is to summarise the diagnostic accuracy of MRI for the identification of root avulsion in adult traumatic BPI. Radiologists and surgeons may use this information to rationalise such imaging, aid in its interpretation and guide future research focused on improving imaging of BPI.

The two objectives are:

1. To determine the diagnostic accuracy of MRI for detecting root avulsion(s) in adults with traumatic BPI;
2. To determine the diagnostic accuracy of MRI for detecting pseudomeningocolele(s) as a surrogate marker of root avulsion in adults with traumatic BPI.

## Methods

This protocol was written in accordance with guidance in the Cochrane Handbook for Reviews of Diagnostic Test Accuracy [37] and PRISMA checklist (Additional file 1) and was registered on the PROSPERO database (CRD42016049702).

### Types of studies

We will include all studies of adults with traumatic BPI that report the findings of pre-operative MRI and surgical exploration of the roots of the brachial plexus. We will exclude case reports.

### Participants

This review will include studies concerning patients aged 18 years and over, with symptomatic BPI sustained as a result of non-penetrating trauma. The condition typically affects young men involved in road traffic accidents. All such patients are managed in tertiary (or rarely secondary) care hospitals with expertise in the management of adult BPI. We will exclude those with bilateral injuries as they are a rare subgroup of patients, with very high energy injuries of atypical mechanism, who may have unusual anatomy [38, 39].

### Clinical setting

We will consider any setting.

### Target condition

The target condition is avulsion of the root(s) of the brachial plexus. We acknowledge that different patterns of root avulsion have important clinical differences, such that a single root avulsion (e.g. C5 alone) is clinically different to three-root avulsions or a pan-plexus injury. Nonetheless, we are interested in the ability of MRI to distinguish between normal roots (no root avulsion) and abnormal roots (any root avulsion). Any avulsion injury is important to detect by pre-operative imaging, but the ability of MRI to correctly identify patients with no root avulsion is of paramount importance because exploratory surgery could be avoided in some cases.

### Index test

The role of MRI is to detect root avulsions. This scan is typically used pre-operatively between the time of injury and nerve surgery which could be anytime from the day of injury up to years later. Typically, it is performed within a few weeks following injury.

Several factors vary between MRI scanners including brand (e.g. Siemens versus General Electric) and model, field strength (e.g. a 3 Tesla system is more powerful so it can generate a better image than a 1.5 Tesla system), device software (customisation is different between brands, so too are the postprocessing options), coil arrangement and bandwidth, scanner bore (the smaller the bore the greater the signal generated), gradients (more efficient gradients can provide better images), etc. Therefore, there are important physical variations between scanners which we plan to investigate in this review.

The interpretation of MRI for root avulsion is difficult. The diagnosis is subjective because the judgement of a positive or negative MRI result is made by the radiologist examining the images. MR images are typically reviewed by a radiologist with specialist training in musculoskeletal and neurological imaging. An MR image may be considered positive for root avulsion when there is a perceived lack of continuity or absence of the nerve root between the spinal cord attachment and the exit foramen. The normal nerve root takes an oblique course (from posterior to anterior and cranial to caudal) from the spinal cord to the exiting intervertebral foramina. Therefore, MRI can also be considered positive for avulsion if there is an abnormal course/position of the nerve root because if a root has been avulsed proximally from the spinal cord, then it will descend in the cerebrospinal fluid (CSF) space, adopting a more caudal and horizontal position adjacent to the foramina. Overall, the diagnosis of root avulsion is binary (present or absent), and any permutation of these features may lead a radiologist to make a decision of MRI positivity.

Alongside the above findings, MR images are routinely examined for the presence of a pseudomeningocoele (sometimes erroneously termed a meningocoele). A pseudomeningocoele is defined as an expansion of the space containing the nerve root and CSF within the intervertebral foramen and is typically associated with an abnormal contour of the dura within the spinal canal where dural leaks occur. Occasionally, the leak of CSF extends beyond the foramen into a cystic collection lying in the paraspinal soft tissues, and this too is contained within the definition. Pseudomeningocoeles have been popularised as surrogate markers of root avulsion because rupture of the dura mater is believed to correspond to rupture of the nerve root; however, this is not a reliable sign of true root avulsion as the agreement is moderate at best and again, conflicting across studies [19, 36, 40–42].

Root avulsion or peudomeningocoele can be observed at any spinal level which may affect the brachial plexus, from C4 to T2. Given that BPI are usually unilateral, but have been reported as bilateral, there are potentially 14 spinal levels to comment upon. This is largely irrelevant, as the most important feature of the test is to identify non-cases (true negatives) because if such individuals also did not have post-ganglionic injuries, then exploratory surgery would not be required. Therefore, for the purposes of this review, we consider one suspected avulsion as important as any frequency because any avulsion would warrant surgery (in the form of a nerve transfer) so there would be little morbidity reduction.

### Prior tests

As part of their clinical care in a major trauma centre, patients would be routinely assessed by healthcare professionals for symptoms and signs of major trauma, and specifically for major nerve or spinal cord injury. Similarly, most patients would undergo both plain radiography and computed tomography of injured body parts and will be screened for other injuries (e.g. fractures, vascular injuries, etc.). All such a priori test results would usually be available to the radiologist interpreting the MRI scan specifically to indicate the injured side and possible location of neurological deficit.

### Reference standard

The reference standard for diagnosing root avulsion of the brachial plexus is operative exploration. This involves an operation under general anaesthesia. The incision is in the supraclavicular fossa and may extend to the deltopectoral groove. The roots of the brachial plexus are visualised from C4 (for the pre-fixed plexus [43]) through to T1 as they emerge between the scalene muscles. Some surgeons perform hemilaminectomy of C5-C7 inclusive to visualise the intraforaminal and intradural roots.

Additional intraoperative tests may be used to supplement the reference standard and so help to determine continuity between the spinal cord and roots of the brachial plexus, including somatosensory evoked potentials (SEPs) and bipolar motor nerve stimulation. SEPs involves the application of monophasic square pulses of < 300 ms of varying frequency to the roots of the brachial plexus; a healthy nerve in continuity with the spinal cord will transmit this action potential to the cerebral cortex, which can then be measured using a filtered electroencephalogram but an injured or avulsed nerve will not transmit. Intraoperative nerve stimulators/locators are biphasic instruments which apply a low current (< 20 mA) across a section of nerve; a healthy nerve will induce an action potential and the innervated muscle will contract but no end-organ effect will be observed for an injured nerve. These electrophysiological tests can only be performed intraoperatively and provide supplementary information, and as such, we consider them as part of the reference standard of ‘operative exploration’.

### Search strategy

Electronic database search strategies are available in Additional file 2. We will search MEDLINE, Embase and the Cochrane Library from inception with no restrictions. Using Mendeley reference manager, citations will be imported and de-duplicated. Two authors will independently perform reference tracking to identify potentially relevant studies from the citations of included articles.

### Study selection

Two review authors will independently screen titles and abstracts for relevance, in accordance with the eligibility criteria. The full text of potentially eligible articles will be obtained and independently assessed by two review authors using our full text screening form (Additional file 3). Disagreements between the reviewer authors will be resolved by discussion and consensus or by consulting a third review author. Reasons for exclusion will be recorded. Eligible articles will be imported to Review Manager® version 5 (The Nordic Cochrane Centre, The Cochrane Collaboration) and categorised as included or excluded, for later analyses.

### Data extraction

Two review authors will independently extract data. We will contact the study authors by email or phone if information is missing or unclear. Data extracted will include the following: study identifier; country of origin; number of participants, gender and age; the brand, model and field strength of MRI scanner used; full details of the pulse sequences used; whether intravenous contrast was used; and data for construction of 2 × 2 tables of the number of true positives, false positives, false negatives and true negatives. Disagreements between the review authors will be resolved by consensus or discussion with a third review author if consensus cannot be reached.

### Methodological quality assessment

We will assess the risk of bias and applicability of the included studies using a tailored version of the Quality Assessment of Diagnostic Accuracy Studies (revised tool) (QUADAS-2) [44] (Additional file 4). The strength of the body of evidence will be assessed using the Grades of Recommendation, Assessment, Development and Evaluation (GRADE) tool [45]. Two review authors will perform the assessment independently.

Any discrepancies between review authors will be resolved by consensus or consultation with a third review author. The results of the assessment will be summarised graphically or in a table.

### Data synthesis

We will perform the analyses separately for each target condition. For preliminary analyses and visual assessment of heterogeneity, we will plot estimates of sensitivity and specificity from the included studies on forest plots and in receiver operating characteristic space. If data are sufficient and appropriate to be pooled, we will use a bivariate model for meta-analysis to obtain summary sensitivities and specificities (summary points) [37, 46, 47].

If data permits, we will investigate heterogeneity in the performance of MRI by using subgroup analyses or meta-regression. Meta-regression will be performed by including a variable of interest as a covariate in a bivariate model. As indicated in the index test section, we will investigate an important physical variation between scanners, the field strength. We will perform sensitivity analyses to examine the impact of high or unclear risk of bias in the domains of the QUADAS-2 tool on our findings. We do not plan to assess publication bias because the determinants of publication bias are not well understood for diagnostic accuracy reviews [37] and the Deeks’ test has low power when there is heterogeneity as is typically observed in diagnostic accuracy reviews [48].

We will use Stata version 15 (Stata-Corp, College Station, TX, USA) for the meta-analyses. For generating forest plots as well as summary receiver operating characteristic plots showing summary points along with 95% confidence regions, we will use Review Manager® version 5 (The Nordic Cochrane Centre, The Cochrane Collaboration).

## Discussion

This review will summarise the diagnostic accuracy of MRI for the identification of root avulsion in traumatic BPI in adults. The outcome data may inform future research (focussed on improving shortfalls in the imaging), explain discrepancies between studies and ultimately help to improve the imaging of major nerve injuries.

There are many potential sources of bias in the studies of diagnostic accuracy [49]. We expect a minority of patients with BPI will not have undergone operative exploration for various reasons, e.g. they did not consent to surgery, anaesthesia was unsafe or the treatment of other injuries took precedence. This would upwardly bias the sensitivity of MRI if the proportion of false negatives is underestimated. This problem cannot be reliably mitigated by replacing or supplementing exploratory surgery with another reference standard, e.g. clinical observation because (a) the reanimation of the limb may evolve over several years and concurrent stiffness and contractures may prevent reliable and repeatable inferences about function; (b) testing a given muscle in isolation (and thus the supplying root) may be impossible as many movements involve several muscles working in synergy, each receiving input from different nerves and thus, different cervical roots; (c) cerebral cortical reorganisation may undermine clinical observations of muscle power and sensation, e.g. where two roots supply one muscle and one is injured, the cortex may reroute more input to the remaining fibres, confounding the assessment of function; and (d) the end organs of the limb (muscles, skin, joints, tendons, etc.) may receive input from several roots and equally, one root may innervate several structures, so the constellation of clinical abnormalities cannot be confidently attributed to a specific site of injury. In contrast, the diagnostic accuracy of MRI may be downwardly biased because patients may have been referred based on MRI findings rather than the presence of symptoms alone. We also expect most studies to be retrospective, and some studies may have recruited an unrepresentative sample of patients which may bias diagnostic accuracy and raise concerns about applicability. These and other issues will be addressed in the quality assessment, and we will interpret the findings of our review with respect to these potential limitations.

## Additional files

Additional file 1: PRISMA-P checklist. (DOC 82 kb)

Additional file 2: Search strategies. (DOCX 17 kb)

Additional file 3: Screening form. (DOCX 23 kb)

Additional file 4: QUADAS-2 tool. (DOCX 18 kb)

## Abbreviations

BPI: Brachial plexus injury
CSF: Cerebrospinal fluid
DTA: Diagnostic test accuracy
GRADE: Grades of Recommendation, Assessment, Development and Evaluation
MRI: Magnetic resonance imaging
NIHR: National Institute for Health Research
PRISMA: Preferred Reporting Items for Systematic Reviews and Meta-analyses
QUADAS-2: Quality Assessment of Diagnostic Accuracy Studies
SEP: Somatosensory evoked potential

## References

[1] National Audit Office. Major Trauma Care in England. 2010. https://www.nao.org.uk/wp-content/uploads/2010/02/0910213.pdf.

[2] Midha R (1997). Epidemiology of brachial plexus injuries in a multitrauma population. Neurosurgery.

[3] Franzblau LE, Shauver MJ, Chung KC (2014). Patient satisfaction and self-reported outcomes after complete brachial plexus avulsion injury. J Hand Surg Am.

[4] Kretschmer T, Ihle S, Antoniadis G, Seidel JA, Heinen C, Börm W (2009). Patient satisfaction and disability after brachial plexus surgery. Neurosurgery.

[5] Dolan RT, Butler JS, Murphy SM, Hynes D, Cronin KJ (2012). Health-related quality of life and functional outcomes following nerve transfers for traumatic upper brachial plexus injuries. J Hand Surg Eur Vol.

[6] Maldonado AA, Kircher MF, Spinner RJ, Bishop AT, Shin AY (2016). Free functioning gracilis muscle transfer versus intercostal nerve transfer to musculocutaneous nerve for restoration of elbow flexion after traumatic adult brachial pan-plexus injury. Plast Reconstr Surg.

[7] Teixeira MJ, da Paz MG, Bina MT, Santos SN, Raicher I, Galhardoni R (2015). Neuropathic pain after brachial plexus avulsion—central and peripheral mechanisms. BMC Neurol.

[8] Lee KJ, Joo WI, Chough CK, Park HK, Rha HK (2012). Long term effect of thalamic deep brain stimulation for pain due to brachial plexus injury. Stereotact Funct Neurosurg.

[9] Brill S, Aryeh IG (2008). Neuromodulation in the management of pain from brachial plexus injury. Pain Physician.

[10] Franzblau L, Chung KC (2015). Psychosocial outcomes and coping after complete avulsion traumatic brachial plexus injury. Disabil Rehabil.

[11] Wilson TJ, Chang KWC, Yang LJ-S (2016). Depression and anxiety in traumatic brachial plexus injury patients are associated with reduced motor outcome after surgical intervention for restoration of elbow flexion. Neurosurgery.

[12] Mancuso CA, Lee SK, Dy CJ, Landers ZA, Model Z, Wolfe SW (2015). Expectations and limitations due to brachial plexus injury: a qualitative study. Hand.

[13] Liu Y, Lao J, Gao K, Gu Y, Zhao X (2013). Functional outcome of nerve transfers for traumatic global brachial plexus avulsion. Injury.

[14] Vargas MI, Viallon M, Nguyen D, Beaulieu JY, Delavelle J, Becker M (2010). New approaches in imaging of the brachial plexus. Eur J Radiol.

[15] Nardin RA, Patel MR, Gudas TF, Rutkove SB, Raynor EM (1999). Electromyography and magnetic resonance imaging in the evaluation of radiculopathy. Muscle Nerve.

[16] Zhu Y-S, Mu N-N, Zheng M-J, Zhang Y-C, Feng H, Cong R (2014). High-resolution ultrasonography for the diagnosis of brachial plexus root lesions. Ultrasound Med Biol.

[17] Mallouhi A, Meirer R, Bodner G (2003). Sonographic features of brachial plexus traumatic rupture. J Neurosurg.

[18] Lapegue F, Faruch-Bilfeld M, Demondion X, Apredoaei C, Bayol MA, Artico H (2014). Ultrasonography of the brachial plexus, normal appearance and practical applications. Diagn Interv Imaging.

[19] Sureka J, Cherian RA, Alexander M, Thomas BP (2009). MRI of brachial plexopathies. Clin Radiol.

[20] Karalija A, Novikova LN, Orädd G, Wiberg M, Novikov LN (2016). Differentiation of pre- and postganglionic nerve injury using MRI of the spinal cord. PLoS One.

[21] Ruven C, Chan TK, Wu W (2014). Spinal root avulsion: an excellent model for studying motoneuron degeneration and regeneration after severe axonal injury. Neural Regen Res.

[22] Dubuisson AS, Kline DG (2002). Brachial plexus injury: a survey of 100 consecutive cases from a single service. Neurosurgery.

[23] Palazzi S, Bonnard C, Raimondi P. Symposium on brachial plexus surgery. A Narakas Club. Barcelona (Spain)--13 and 14 March 1999. Chir Main 1999;18:167–171. 10885964

[24] Nagano A (1998). Treatment of brachial plexus injury. J Orthop Sci.

[25] Terzis JK, Kostopoulos VK (2007). The surgical treatment of brachial plexus injuries in adults. Plast Reconstr Surg.

[26] Siqueira MG (2011). Surgical treatment of adult traumatic brachial plexus injuries: an overview. Arq Neuropsiquiatr.

[27] Tung TH, Mackinnon S (2003). Brachial plexus injuries. Clin Plast Surg.

[28] Jivan S, Novikova LN, Wiberg M, Novikov LN (2006). The effects of delayed nerve repair on neuronal survival and axonal regeneration after seventh cervical spinal nerve axotomy in adult rats. Exp Brain Res.

[29] Jivan S, Kumar N, Wiberg M, Kay S (2009). The influence of pre-surgical delay on functional outcome after reconstruction of brachial plexus injuries. J Plast Reconstr Aesthetic Surg.

[30] Simon NG, Spinner RJ, Kline DG, Kliot M (2015). Advances in the neurological and neurosurgical management of peripheral nerve trauma. J Neurol Neurosurg Psychiatry.

[31] Eggers R, Tannemaat MR, De Winter F, Malessy MJA, Verhaagen J (2016). Clinical and neurobiological advances in promoting regeneration of the ventral root avulsion lesion. Eur J Neurosci.

[32] Fournier HD, Mercier P, Menei P (2005). Repair of avulsed ventral nerve roots by direct ventral intraspinal implantation after brachial plexus injury. Hand Clin.

[33] Kachramanoglou C, De Vita E, Thomas DL, Wheeler-Kingshott CA, Balteau E, Carlstedt T (2013). Metabolic changes in the spinal cord after brachial plexus root re-implantation. Neurorehabil Neural Repair.

[34] Tagliafico A, Succio G, Serafini G, Martinoli C (2012). Diagnostic accuracy of MRI in adults with suspect brachial plexus lesions: a multicentre retrospective study with surgical findings and clinical follow-up as reference standard. Eur J Radiol.

[35] Tsai P-Y, Chuang T-Y, Cheng H, Wu H-M, Chang Y-C, Wang C-P (2006). Concordance and discrepancy between electrodiagnosis and magnetic resonance imaging in cervical root avulsion injuries. J Neurotrauma.

[36] Yoshikawa T, Hayashi N, Yamamoto S, Tajiri Y, Yoshioka N, Masumoto T (2006). Brachial plexus injury: clinical manifestations, conventional imaging findings, and the latest imaging techniques. Radiographics.

[37] Collaboration TC. The Cochrane Handbook for Diagnostic Test Accuracy Reviews. 2016. http://methods.cochrane.org/sdt/handbook-dta-reviews.

[38] Sambasivan A, Pandit S (2013). Bilateral brachial plexus injury after a hanging suicide attempt: a case report. PM&R.

[39] Anwar F, McLaughlin D (2012). Bilateral brachial plexus injury. J College PhysSurg Pakistan.

[40] Aralasmak A, Karaali K, Cevikol C, Uysal H, Senol U (2010). MR imaging findings in brachial plexopathy with thoracic outlet syndrome. Am J Neuroradiol.

[41] van Es HW, Bollen TL, van Heesewijk HPM (2010). MRI of the brachial plexus: a pictorial review. Eur J Radiol.

[42] Doi K, Otsuka K, Okamoto Y, Fujii H, Hattori Y, Baliarsing AS (2002). Cervical nerve root avulsion in brachial plexus injuries: magnetic resonance imaging classification and comparison with myelography and computerized tomography myelography. J Neurosurg.

[43] Pellerin M, Kimball Z, Tubbs RS, Nguyen S, Matusz P, Cohen-Gadol AA (2010). The prefixed and postfixed brachial plexus: a review with surgical implications. Surg Radiol Anat.

[44] Whiting P, Rutjes AWS, Reitsma JB, Bossuyt PMM, Kleijnen J (2003). The development of QUADAS: a tool for the quality assessment of studies of diagnostic accuracy included in systematic reviews. BMC Med Res Methodol.

[45] Atkins D, Best D, Briss PA, Eccles M, Falck-Ytter Y, Flottorp S (2004). Grading quality of evidence and strength of recommendations. BMJ.

[46] Chu H, Cole SR (2006). Bivariate meta-analysis of sensitivity and specificity with sparse data: a generalized linear mixed model approach. J Clin Epidemiol.

[47] Reitsma JB, Glas AS, Rutjes AWS, Scholten RJPM, Bossuyt PM, Zwinderman AH (2005). Bivariate analysis of sensitivity and specificity produces informative summary measures in diagnostic reviews. J Clin Epidemiol..

[48] Deeks JJ, Macaskill P, Irwig L (2005). The performance of tests of publication bias and other sample size effects in systematic reviews of diagnostic test accuracy was assessed. J Clin Epidemiol.

[49] Rutjes AWS (2006). Evidence of bias and variation in diagnostic accuracy studies. Can Med Assoc J.

