# Supplementary material for: Magnetic resonance imaging for detecting root avulsions in traumatic adult brachial plexus injuries: protocol for a systematic review of diagnostic accuracy
Source: Syst Rev. 2018 May 19;7:76. doi: 10.1186/s13643-018-0737-2 (PMC5960500; doi:10.1186/s13643-018-0737-2)
Supplement: Supplementary file 2 — Search strategies. (DOCX 17 kb) [file 13643_2018_737_MOESM2_ESM.docx]

**Brachial Plexus Injury MRI DTA review Search Strategy**

**Medline (1946 to current date)**

1. exp brachial plexus (tree A08.800.800.720.050)
2. (brachial AND plexus).ti,ab
3. (root AND avulsion).ti,ab
4. (pre?ganglion*).ti,ab
5. ((brachial AND plexus) AND pseudomeningocoele).ti,ab
6. exp magnetic resonance imaging (tree E01.370.350.825.500)
7. (MR?).ti,ab
8. (magnetic AND resonance).ti,ab
9. (NMR).ti,ab
10. (neurogra*).ti,ab
11. (DTI).ti,ab
12. ((diffusion AND tensor) AND imaging).ti,ab
13. (1 OR 2 OR 3 OR 4)
14. (5 OR 6 OR 7 OR 8 OR 9 OR 10 OR 11 OR 12)
15. (13 AND 14)

**EMBASE (1947 to current date)**

1. exp brachial plexus
2. (brachial AND plexus).ti,ab
3. (root AND avulsion).ti,ab
4. (pre?ganglion*).ti,ab
5. ((brachial AND plexus) AND pseudomeningocoele).ti,ab
6. exp magnetic resonance imaging
7. (MR?).ti,ab
8. (magnetic AND resonance).ti,ab
9. (NMR).ti,ab
10. (neurogra*).ti,ab
11. (DTI).ti,ab
12. ((diffusion AND tensor) AND imaging).ti,ab
13. (1 OR 2 OR 3 OR 4)
14. (5 OR 6 OR 7 OR 8 OR 9 OR 10 OR 11 OR 12)
15. (13 AND 14)

**Cochrane Library**

“brachial plexus” with no limitations
