# Supplementary material for: Magnetic resonance imaging for detecting root avulsions in traumatic adult brachial plexus injuries: protocol for a systematic review of diagnostic accuracy
Source: Syst Rev. 2018 May 19;7:76. doi: 10.1186/s13643-018-0737-2 (PMC5960500; doi:10.1186/s13643-018-0737-2)
Supplement: Supplementary file 3 — Screening form. (DOCX 23 kb) [file 13643_2018_737_MOESM3_ESM.docx]

**Full text screening form**

**Study ID:**

| **Study characteristic** | **Decision** (Yes / No / Unclear) |
| --- | --- |
| What is the study design? | |
| Cross sectional study |  |
| Case control study |  |
| Case series |  |
| Prospective or retrospective cohort study |  |
| Randomised controlled trial |  |
| Systematic review |  |
| Other (e.g. narrative review) |  |
| Are the participants adults with traumatic brachial plexus injury? |  |
| Is the index test pre-operative MRI? |  |
| Is the reference standard surgical exploration of the supraclavicular brachial plexus? |  |
| Did the study report measures of test accuracy? | |
| Sensitivity and specificity |  |
| Predictive values |  |
| Likelihood ratios |  |
| ROC curve/AUC |  |
| Overall diagnostic accuracy |  |
| Is it possible to derive a 2x2 table? |  |

For a study to be included it must meet the following criteria:

1. Primary study
2. Participants are adults with traumatic brachial plexus injury
3. Index test was MRI
4. Reference standard was surgical exploration of the supraclavicular brachial plexus
5. Includes data on diagnostic accuracy
6. Possible to derive 2x2 table of number of true positives, false positives, false negatives and true negatives

**Overall decision:** Include Exclude Unclear
