# Supplementary material for: Magnetic resonance imaging for detecting root avulsions in traumatic adult brachial plexus injuries: protocol for a systematic review of diagnostic accuracy
Source: Syst Rev. 2018 May 19;7:76. doi: 10.1186/s13643-018-0737-2 (PMC5960500; doi:10.1186/s13643-018-0737-2)
Supplement: Supplementary file 4 — QUADAS-2 tool. (DOCX 18 kb) [file 13643_2018_737_MOESM4_ESM.docx]

**QUADAS-2: Magnetic Resonance Imaging for Detecting Root Avulsions in Traumatic Brachial Plexus Injuries in Adults**

**Study ID:**

*Patients (setting, intended use of index test, presentation, prior testing):*

*Index test(s):*

*Reference standard and target condition:*

**1 – Patient Selection**

1A. Risk of Bias

Describe methods of patient selection:

Was a consecutive or random sample of patients enrolled? Yes / No / Unclear

Was a case-control design avoided? Yes / No / Unclear

Did the study avoid inappropriate exclusions (eg. unclear Yes / No / Unclear

avulsion status at surgery, older patients, etc)?

**Could the selection of patients have introduced bias? LOW / HIGH / UNCLEAR**

*Code low risk if the answers to all signalling questions were yes.*

*Code as high risk if any answer to the signalling questions was no.*

*Otherwise, code as unclear.*

1B. Concerns regarding applicability

Describe included patients (prior testing, presentation, intended use of index test and setting)

**Is there concern that the included patients do not**

**match the review question? LOW / HIGH / UNCLEAR**

*Code as unclear concern if any of the following were not described: prior tests, mechanisms of injuries, the role and of the MRI and conditions (pulse sequence, interpretation, etc) or surgical method of exploration.*

*Code as low concern if any above factors were described and appropriate.*

*Code as high concern if: >1 MRI was performed preoperatively or multiple surgical explorations were performed by different parties.*

**2: Index test**

2A. Risk of Bias

Describe the index test and how it was conducted and interpreted

Were the MRI results interpreted without knowledge of the exploration? Yes / No / Unclear

If a threshold for either test was used, was it pre-specified Yes / No / Unclear

*Code as low risk if both the diagnosis of avulsion was defined for the MRI and exploratory surgery*

*Code as high risk the diagnosis of avulsion was not described for the MRI or reference standard*

*Otherwise, code as unclear.*

**Could the conduct or interpretation of the index**

**test have introduced bias? LOW / HIGH / UNCLEAR**

*Code as low risk if all the signalling questions were answered yes.*

*Code as high risk if all signalling question were answered no.*

*Otherwise, code as unclear.*

2B. Concerns regarding applicability

**Is there concern that the index test, its conduct, or**

**interpretation differ from the review question? LOW / HIGH / UNCLEAR**

Code as high concern if images were not interpreted by an experienced radiologist or explorations were not carried out by an appropriately trained and experienced surgeon or MRIs were interpreted by multiple radiologists, as this does not reflect practice.

Code as low concern if both tests were performed by appropriate personnel.

Otherwise, code as unclear concern if MRI images were.

**3: Reference Standard**

3A. Risk of Bias

Describe the reference standard and how it was conducted and interpreted

Is the reference standard likely to correctly classify the target condition? Yes/No/Unclear

Were the reference standard results interpreted without knowledge of the

results of the index test? Yes/No/Unclear

**Could the reference standard, its conduct, or its**

**Interpretation have introduced bias? LOW / HIGH / UNCLEAR**

Code as low risk if both signalling questions answer yes.

Code as high risk if both signalling question answers no.

Otherwise code as unclear risk.

3B. Concerns regarding applicability

**Is there concern that the target condition as defined by**

**the reference standard does not match the review question? LOW / HIGH / UNCLEAR**

*Code as unclear concern if the criteria for diagnosis of root avulsion at surgery was unclear or incompletely described in the methods and results are reported.*

*Code as low concern if the criteria for root avulsion at surgery was clearly defined and results reported.*

*Code as high concern if there was no description of the reference standard in the methods and results alone are reported.*

**4 – Flow and Timing**

4A. Risk of Bias

Describe any patients who did not receive a preoperative MRI and/or exploration or who were excluded from the 2x2 table:

Describe the time interval and any interventions between index test(s) and reference standard

Was the interval between MRI and exploration <12 months? Yes / No / Unclear

Did all cases receive the same brachial plexus exploration? Yes / No / Unclear

Were all patients included in the analysis? Yes / No / Unclear

**Could the patient flow have introduced bias? LOW / HIGH / UNCLEAR**

Code as low risk if all signalling questions were answered yes

Code as high risk if any answer was no

Otherwise, code as unclear
